# Induction of Broadly Neutralising HCV Antibodies in Mice by Integration-Deficient Lentiviral Vector-Based Pseudotyped Particles

**DOI:** 10.1371/journal.pone.0062684

**Published:** 2013-04-23

**Authors:** Yao Deng, Jie Guan, Bo Wen, Na Zhu, Hong Chen, Jindong Song, Yang Yang, Yue Wang, Wenjie Tan

**Affiliations:** 1 Key Laboratory of Medical Virology and Viral Diseases, Ministry of Health, National Institute for Viral Disease Control and Prevention, Chinese Center for Disease Control and Prevention, Changping District, Beijing, People’s Republic of China; 2 College of Life Science, Jilin University, Jilin Province, Chang Chun, People’s Republic of China; Public Health Agency of Canada, Canada

## Abstract

**Introduction:**

Integration-deficient lentiviral vectors (IDLVs) are a promising platform for immunisation to elicit both humoral immunity and cellular mediated immunity (CMI). Here, we compared the specific immunity in mice immunised via different regimens (homologous and cocktail) with IDLV-based HCV pseudoparticles (HCVpps) carrying pseudotyped glycoproteins E1E2 and bearing the HCV NS3 gene. Humoral and cell-mediated immune responses were also evaluated after IDLV-HCVpp immunisation combined with heterologous rAd5-CE1E2 priming protocols. Sera from the mice effectively elicited anti-E1, -E2, and -NS3 antibody responses, and neutralised various HCVpp subtypes (1a, 1b, 2a, 3a and 5a). No significant CMI was detected in the groups immunised with IDLV-based HCVpps. In contrast, the combination of rAd5-CE1E2 priming and IDLV-based HCVpp boosting induced significant CMI against multiple antigens (E1, E2, and NS3).

**Conclusion:**

IDLV-based HCVpps are a promising vaccination platform and the combination of rAd5-CE1E2 and IDLV-based HCVpp prime-boost strategy should be further explored for the development of a cross-protective HCV vaccine.

## Introduction

Hepatitis C virus (HCV) infection is a major cause of chronic hepatitis, cirrhosis, and hepatocellular carcinoma [1]. Among HCV-infected individuals, ∼20% will eradicate the virus spontaneously, while the remaining 80% will develop chronic disease [1]. The current treatment for chronic hepatitis C exhibits limited efficacy, adverse effects, a high cost, and impaired cost performance [2]. Thus, a prophylactic vaccine that prevents or attenuates the primary infection and a therapeutic vaccine that increases cure rates for infected patients are of important clinical significance [1]. The development of an HCV vaccine using classical principles is problematic [3], [4]. Along with molecular biomedicine, vaccine development has greatly advanced, and many peptide, protein, DNA, viral-like particle (VLP), and viral vector-based vaccines have reached clinical trials [4]. A viral vector approach has structural biological merits, is convenient for molecular modification in vaccine development, and has shown promising immune responses in many reports [4]. Lentiviral vectors (LVs) can transduce both dendritic cells and other antigen-presenting cells efficiently, resulting in long-term antigen expression and presentation [5]–[7]. LVs are under intense scrutiny as unique candidate viral vector vaccines against tumors and aggressive pathogens due to their ability to initiate potent and durable specific immune responses [7]–[9].

Strategies that alleviate safety concerns will facilitate the practical application of LVs[6], [7]. The development of integration-deficient LVs (IDLVs) may circumvent the safety concerns raised by insertional mutagenesis [10]. IDLVs achieved by integrase mutations could not only prevent proviral integration but also increase the number of circular vector episomes in transduced cells [10]. IDLVs can mediate transient gene expression in proliferating cells, stable expression in non-dividing cells in vitro and in vivo, and specific immune responses [10].

Several studies have emphasized the importance of early and highly neutralising antibody (nAb) responses for the clearance of HCV infections [11]–[15]. However, HCV NS5B lacks a proofreading function, leading to high genetic variability and the avoidance of host immune responses [16]. Six major HCV genotypes and 100 subtypes have been identified worldwide [16]. Thus, a key issue in HCV vaccine development is to find methods that elicit high titres of broadly cross-reactive nAbs [1, 3, and 4]. The inclusion of neutralising epitopes and B cell boosting ability in a vaccine is critical. Normally, viral envelope proteins in their proper conformation displayed on VLPs could achieve the desired effect [17]. Previous work has shown that the E1E2 envelope protein derived from different HCV subtypes can be pseudotyped (HCVpp) on recombinant retroviral vectors or LVs [18]–[20]. Meanwhile, T cell-mediated immunity (CMI) is critical for HCV clearance[1], [3], [4], [21]; studies in both chimpanzees and human subjects demonstrated that an early and sustained cell-mediated immune response against the conserved NS3 antigen is essential for recovery from HCV infection[1], [4], [22].

In this study, various IDLV-based HCVpps were engineered, on which HCV envelope proteins were displayed and NS3 mRNA was embedded within the pp. Humoral and cellular immunity induced by homologous and cocktail regimens consisting of different IDLV-HCVpps were evaluated in mice. Moreover, a vaccination strategy that combined priming with recombinant adenovirus type 5 (rAd5) carrying the HCV structural gene (C-E1-E2) [21] and boosting with IDLV-HCVpps was evaluated.

## Materials and Methods

### Plasmid Construction

The integration-deficient packaging plasmid pCMVΔR8.2D64E was derived from pCMVΔR8.2 (a generous gift from Dr. D. Trono) with a point mutation in the integrase (D64E) domain of human immunodeficiency virus (HIV) [5], [23]. The HCV NS3 gene was inserted into the transfer vector pCS-CG (a generous gift from Dr. I. Verma) [6] to provide IDLV gag-binding NS3 mRNA (pCS-NS3). The envelope plasmid pVRC-E1E2 encoding the HCV E1E2 glycoprotein of HCV subtypes 1a (H77), 1b (Hebei), and 2a (JFH1) was described previously [19], [20], [24]. All plasmid DNA was purified using a Qiagen EndoFree Plasmid Maxi Kit. All constructs were confirmed by sequencing or Western blotting.

### Generation of IDLV-HCVpps and rAd5-C/E1/E2

All IDLV-HCVpps were produced by the transfection of human embryonic kidney (HEK) 293FT cells using Fugen HD reagent (Roche, Basel, Switzerland) with a combination of pCMVΔR8.2D64E, pCS-NS3, and pVRC-E1E2. As a control, pVRC-E1E2 was replaced with pMD.G and pCS-NS3 was replaced with pCS-CG [6]. IDLV-HCVpps were harvested 48 h post-transfection, and the virus titre (presented as the HIV p24 antigen concentration) was determined using the Vironostika HIV-1 Antigen Microelisa System (BioMérieux, Shanghai, China). Recombinant adenovirus rAd5-C/E1/E2 carrying the genes encoding the core and E1E2 glycoproteins of HCV subtype 1b (Hebei strain) was described previously [21].

### Characterisation and Validation of the IDLV-HCVpps

The presence of HIV-p24, E2, and NS3 in the IDLV-HCVpps was assessed by Western blotting. Briefly, IDLV-HCVpps were lysed, separated by 12% polyacrylamide gel electrophoresis and transferred by electroblotting to a polyvinylidene fluoride membrane. The membrane was blocked for 1 h in 5% skim milk at 37°C, and then probed with monoclonal antibodies (mAb) to HCV E2 (AP33) or p24 (clone 183-H12, the AIDS Reagents and Depositary program, NIAID, NIH) overnight at 4°C. After washing three times with PBST (PBS containing 0.5% Tween-20), goat anti-mouse antibodies (IRDye 800) were added and incubated for 1 h at 37°C. After washing three times with PBST, the protein was visualised using an infrared imaging system.

To assess NS3 expression induced by the IDLV-HCVpps, Huh7.5/CD81cells (from Dr. T. Wakita) were collected and lysed 3 or 4 days after infection by the IDLV-HCVpps, and NS3 was detected by Western blotting using mAb against HCV NS3 (Thermo Fisher ABR,USA). Electron microscopy was used to verify the presence of the IDLV-HCVpps.

### Immunisation of Mice

All animal experiments were conducted in accordance with the Guidelines for Animal Experiments described and approved by the Institutional Animal Care and Use Committee (IACUC) of Chinese Center for Disease Control and Prevention. Six or twelve mice (female BalB/c, 6–8 weeks old) per group were immunised intramuscularly using IDLV-HCVpps with adjuvant (Al [OH] 3+cytosine phosphorothioate guanine oligodeoxynucleotide [CpG ODN] 1826). Mice injected with PBS acted as controls.

### Enzyme-linked Immunosorbent Assay (ELISA)

Soluble E1 or E2 glycoprotein comprising a C-terminal His tag were produced by the transient transfection of 293T cells, and quantified as described previously [24]. Purified E1, E2 were coated onto 96-well plates (Corning Inc., Corning, NY) for ELISA [21], [24]. Purified soluble truncate NS3 protein of HCV or p24 antigen (gifts from Wantai Biotech Company, China) were also coated as antigen onto plates to detect the antibody response using ELISA. Sera were diluted serially to determine the IgG titres, defined as the reciprocal of the serum dilution at which the absorbance was twice that of control sera.

### nAb Analysis

To evaluate the nAbs raised by various IDLV-HCVpps and prime-boost regimens, we produced HCVpps (1a, 1b, 2a, 3a and 5a) that harboured a Luciferase reporter gene [25], [26]. Serially-diluted sera after purification with protein G column were incubated with HCVpps, and then the mixtures were added to Huh7 cells for infection. After 48 h, the cells were lysed for a luciferase activity assay. Normalized neutralisation were calculated as: (relative luciferase units of HCVpps with mock sera - relative luciferase units of HCVpps with immune serum in a given dilution)/relative Luciferase units of HCVpps with mock sera.

### Enzyme-linked Immunosorbent Spot (ELISPOT) Assays

Splenocytes (2 to 5×10^5^) were harvested 2 weeks post-immunisation and stimulated with HCV or HIV peptide pools [21, 27, and 28]. Briefly, 96-well plates were coated overnight with 100 µl per well of 5 µg/ml anti-mouse gamma interferon antibodies (IFN-γ) (BD Pharmingen) in PBS. The plates were then washed three times with RPMI 1640 containing 10% FBS, blocked for 2 h with RPMI 1640 containing 10% FBS and incubated with peptide pools and mononuclear spleen cells (MNCs) in triplicate in a 100-µl reaction mixture. The HCV peptide pools used in this study spanned the core, E1, E2, and NS3 proteins and comprised peptides of 13–17 amino acids [21], [27]. The HIV peptide pool of gag or pol proteins was described elsewhere [28]. Stimulation with PMA (50 ng/ml) and ionomycin (1 µg/ml) was used as a positive control to generate and detect antigen-specific T cells by ELISPOT. The plates were stored in a sealed plastic bag in the dark until analysis using an ELISPOT plate reader.

### Statistical Analysis

Significant differences between the experimental and control groups were evaluated using the one-way ANOVA analysis function in the SPSS software package (release 12.1; SPSS Inc., Chicago, IL). Differences were considered significant at p<0.05.

## Results

### Production and Validation of IDLV-HCVpps

**Figure 1:**
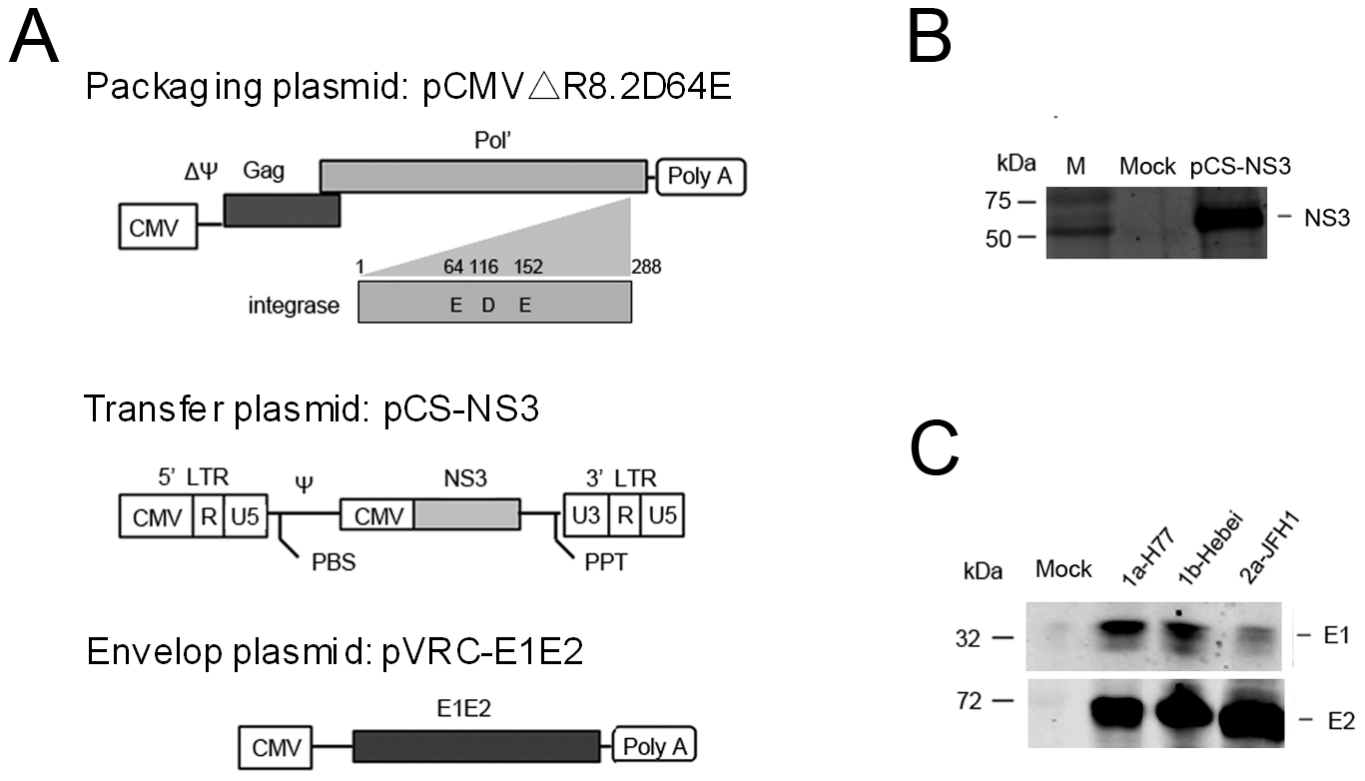
Preparation of immunogens. (**A**) Schematic of the packaging plasmid pCMVΔR8.2D64E, the transfer plasmid pCS-NS3 and the envelope plasmids pVRC-E1E2 The integration-deficient packaging plasmid pCMVΔR8.2D64E transformed from pCMVΔR8.2 with a point mutation in integrase. (**B**) NS3 expression in transfected 293FT cells was detected by Western blotting. (**C**) The expression of E1E2 in 293FT cells transfected with the above plasmids was detected by Western blotting. Each well was loaded with indicated sample (about 10 ng protein) from cell lysates for SDS-PAGE. Primary mAb to HCV NS3, E1, and E2 were applied, and the membranes were probed with goat anti-mouse IgG (IRDye 800). Positive bands were detected using an infrared imaging system.

To improve the safety of the lentivirus-based packaging system, a D64E mutation was introduced into wild-type integrase in the packaging plasmid and named as pCMVΔR8.2D64E (Figure 1A). The mutant was confirmed by sequencing. Plasmid pCS-NS3 contains the HCV NS3-encoding region within the 5′- and 3′-UTRs of self-inactivating HIV-1 transfer plasmid to allow recognition by HIV gag-pol, and allow packaging with the virion. NS3 expression was confirmed by Western blotting using anti-NS3 mAb (Figure 1B). The HCV envelope protein E1E2 expression of different subtypes (1a,1b and 2a) were also constructed and confirmed by Western blotting (Figure 1C) using anti-E1 mAb(A4, a kind gift from J. Dubuisson, Institut de Biologie de Lille, France) and anti-E2 mAb(AP33, a kind gift from from Genentech, Inc.,USA) as the primary antibody, respectively.

**Figure 2:**
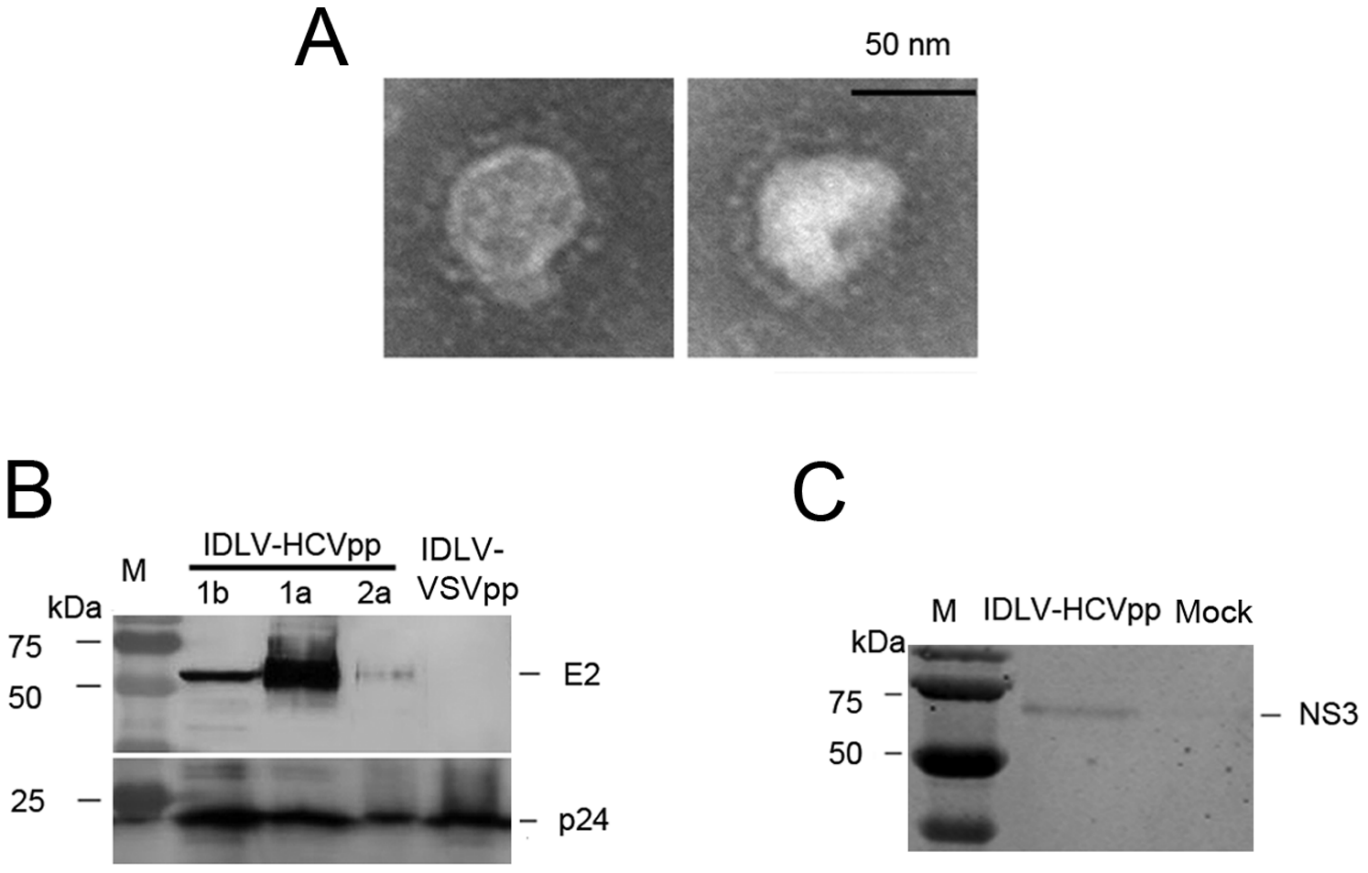
Validation of IDLV-HCVpps. (**A**) The particle structure was revealed for negatively stained IDLV-HCVpps using electron microscopy. (**B**)Expression of E2 and p24 in the IDLV-HCV particles as detected by Western blotting. IDLV-HCVpps were concentrated, lysed, and detected by Western blotting. Primary mAb were detected against E2 and p24. Left lane, low molecular weight protein marker. (**C**)Western blot analyses of NS3 expression in Huh7/CD81 cells infected with IDLV-HCV lentiviral particles. Each well was loaded with indicated sample (about 10 ng protein) from cell lysates for SDS-PAGE. The antibodies and methods were same as above.

IDLV-HCVpps (1a, 1b, and 2a), pseudotyped with HCV E1E2 glycoproteins, and bearing HCV NS3 gene inside the capsids of HIV-1,were produced by transfecting the above three plasmids system into 293T cells. HIV p24 antigen quantity was used to normalise the IDLV-HCVpps. The IDLV-HCVpp structure was observed by electron microscopy (Figure 2A); the particles were 50–100 nm in diameter with envelope protein spikes. To evaluate incorporation of the HCV envelope protein onto the IDLV-HCVpps, normalised IDLV-HCVpps were subjected to Western blotting (Figure 2B). HCV envelope proteins co-existed with p24 in the IDLV-HCVpps. Huh7.5/CD81 cells infected with IDLV-HCVpps were sampled to confirm NS3 expression and the correct tropism by Western blotting (Figure 2C).

### Humoral Immune Responses Rlicited by IDLV-HCVpp Immunisation

**Figure 3:**
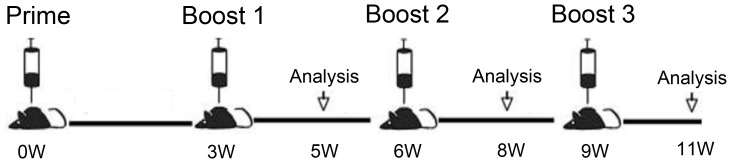
Immunisation schedule of vaccine candidates in this study. Mice were immunized i.m. four times with three weeks apart, respectively. Serum samples from mice were collected two weeks after each boost for IgG detection. Two weeks after the indicated immunization, six mice in each group were sacrificed for ex vivo IFN-γ ELISPOT assays.

The mice were grouped and vaccinated with IDLV-HCVpps or control by various regimens (Figure 3 and Table 1). Immunisation with IDLV-HCVpps induced anti-E1, -E2, -NS3, and -p55 antibody responses after the first boost, and the titres of the above four antibodies increased with enhanced boosting and doses.

**Table 1:** Immunization program.

Group	Immunogens and Dose (per mouse)							
A	PBS	100 µl	PBS	100 µl	PBS	100 µl	PBS	100 µl
B	IDLV-HCVpp (1b)	200 ng p24	IDLV-HCVpp (1b)	200 ng p24	IDLV-HCVpp (1b)	600 ng p24	IDLV-HCVpp (1b)	800 ng p24
C (cocktail)	IDLV-HCVpp(1a+1b+2a)		IDLV-HCVpp(1a+1b+2a)		IDLV-HCVpp(1a+1b+2a)		IDLV-HCVpp(1a+1b+2a)	
D (prime boost)	rAd5-CE1E2	5×10^9^ vp	IDLV-HCVpp (1b)		IDLV-HCVpp (1b)		IDLV-HCVpp (1b)	

**Figure 4:**
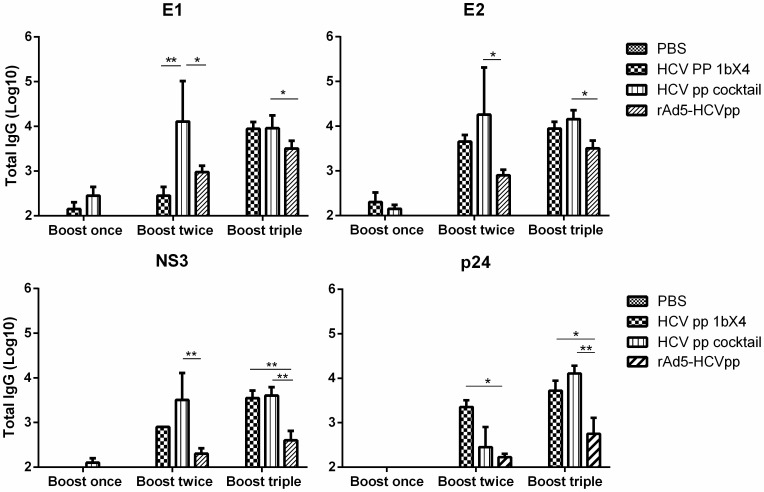
Antigen specific IgG antibody detected by ELISA. Plates were coated with purified soluble E1 or E2 glycoprotein or NS3 of HCV as antigen to detect the specific humoral response against HCV. P24 antigen derived from HIV-1 was also coated as antigen to detect the antibody response against gag protein of lentiviral vector. Sera obtained 2 weeks after the indicated immunisation from each group and tested at a serial dilution. Bars represent the mean of six individual sera of each group; error bars represent the SEM. Significant p-values between the vaccinated groups are shown. *p<0.05, **p<0.01.

**Figure 5:**
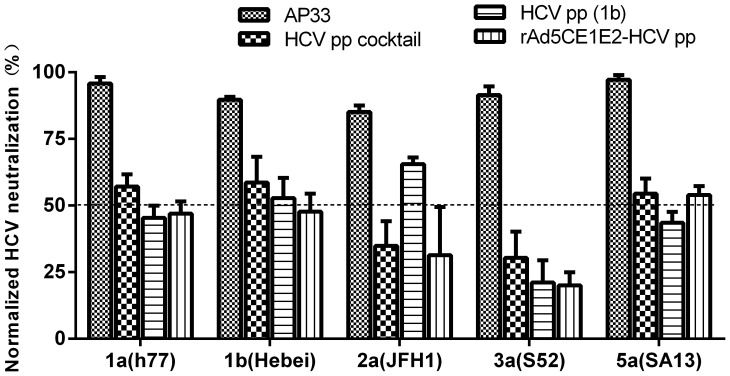
IDLV-HCVpps elicit antibody responses that cross-neutralise HCVpps. Sera obtained 2 weeks after the final immunisation were purified with protein G column and tested at a single dilution of 1∶200 for the neutralisation of HCVpps expressing diverse envelope glycoproteins. AP33 antibody (1∶400 dilutions) was used as positive control and sera from PBS immunization as mock. The percent neutralisation was determined by comparing the infectivity (luciferase relative light units, RLU) of HCVpp-H77 (subtype 1a), Hebei (subtype 1b), JFH1 (subtype 2a), S52 (subtype 3a), and SA13 (subtype 5a) in the presence of test immune sera to the infectivity in the presence of control pre-immune sera at the same dilution. The data are presented as the normalized HCV neutralising inhibition rate (%) at a 1∶200 dilution of sera from the indicated group. Bars represent the mean of six individual sera of each group; error bars represent the SEM.

In the homologous immunisation groups(Figure 4), the E1 antibody titres induced by immunisation was lower than the cocktail group after the second boost with a low IDLV-HCVpp dose (200 ng p24/dose). However, after the triple boost with 800 ng p24/dose, the anti- E1, -E2, and -NS3 antibody titres in both groups increased to an similar level. In addition, the anti- E1, -E2, and -NS3 antibody titres as well as anti-vector (p24) titre in HCVpp cocktail group was significantly higher than the rAd-HCVpp group (*P*<0.05, or *P*<0.001).In terms of nAbs, sera from mice immunised with homologous (1b genotype) or cocktailed (1a+1b+2a) IDLV-HCVpps triple boosts could not only neutralise homologous HCVpps (1b, Hebei) up to 50% at a 1∶200 dilution but also cross-neutralise heterologous HCVpps (1a, 2a, 3a and 5a) from 20–60% at a 1∶200 dilution, and cross neutralizing ability against 3a induced by immunisation were lower than those against HCV of other genotypes tested (Figure 5).

No detectable secretion of IFN-γto HCV antigens (E1, E2, or NS3) was detected by ELISPOT (<50 SFC/10^6^ MNCs) in groups B and C after triple IDLV-HCVpp immunisation only(data not shown).

### IDLV-HCVpp Immunogenicity is Significantly Improved by rAd Priming

**Figure 6:**
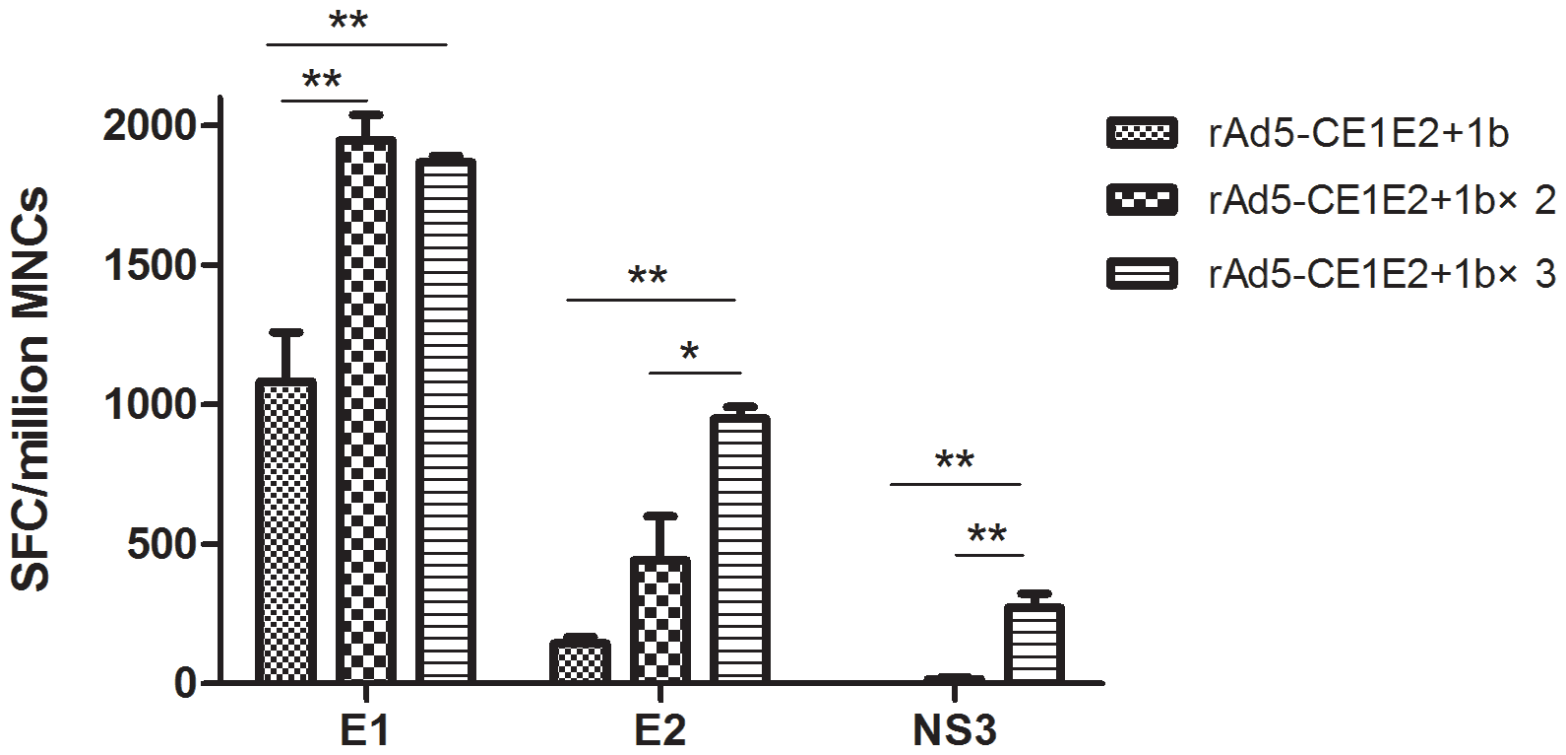
Specific cellular immune responses against HCV immunogens in the rAd5-CE1E2 primed and IDLV-HCV boosted immunisation groups. CMI detected by an ELISPOT assay. ELISPOT assay of splenocytes after stimulation with HCV peptide pools E1, E2, and NS3 representing E1 protein or the C-terminus of E2 or NS3 and the HIV peptides gag and pol. The data are expressed as spot forming cell (SFC) responses to different peptide pools and presented as the mean with SEM for six mice per group. Significant p-values between the vaccinated groups are shown. The control group (received IVDC-VSV) generated SFC responses of <20 per 10^6^ mononuclear cells. Significant p-values between the vaccinated groups are shown. *p<0.05, **p<0.01.

To determine whether the heterologous prime-boost strategy could further enhance the immunogenicity of IDLV-HCVpps in mice, we generated an adenovirus (Ad) –based recombinant vectors expressing structural protein(C/E1/E2) of HCV (1b, subtype) as previous described [21], then used in prime-boost regimen. We compared the immune response characteristics of IDLV-HCVpp boosting combined with a rAd5-CE1E2 HCV vaccine candidate priming. A combination recombinant viral vector rAd5-CE1E2 prime and IDLV- HCVpp boost regimen in this study induced significantly CMI and IgG antibody against multiple target antigens (E1, E2, and NS3), which gradually increased with multiple IDLV- HCVpp boosts (Figure 4 and Figure 6). IDLV-HCVpps triple boosts could not only neutralise homologous HCVpps but also cross-neutralise heterologous HCVpps (1a, 2a, 3a and 5a) from 20–60% at a 1∶200 dilution (Figure 5).

Although the above experiments show that the IDLV-HCVpps alone did not markedly trigger CMI, when IDLV-HCVpp boosting was combined with rAd5-CE1E2 priming, a robust cell-mediated immune response to E1 and E2 was detected after first IDLV-HCVpp boosting, and were significantly enhanced after the third IDLV-HCVpp boost(Figure 6). Notably, an NS3-specific cell-mediated immune response was also detected after triple boosting with IDLV-HCVpps. However, little spots(less than 50) were detected after stimulation with HIV-1 gag and pol (data not shown). This indicates that the CMI to HCV proteins was stimulated significantly by IDLV-HCVpp when priming with rAd5-CE1E2.

These results suggest that IDLV-HCVpps significantly enhanced and broadened the nAb response when combined with rAd5-CE1E2 priming.

## Discussion

The aim of this study is to develop a vaccine platform that specifically trigger both board nAb and CMI against HCV. IDLV represents a promising approach to vaccine development against viral diseases and cancer [10], [29]. We report here the development and validation of IDLV-HCVpps, then to study the capacity of to generate protective immunity against HCV in mice, HCV envelope proteins were displayed on the surface of IDLV virions; Furthermore, The coding sequence of HCV NS3, which harboured critical T cell epitopes for HCV clearance, was inserted into the IDLV virions. Homologous immunisation with the IDLV-HCVpps elicited a robust humoral immune response, including nAbs and IgG antibodies against NS3 and the envelope protein. Sequential and cocktail immunisation with the IDLV-HCVpps further enhanced this response. Priming with rAd5-CE1E2 and boosting with the IDLV-HCVpps elicited strong humoral and T cell-mediated responses.

Numerous studies of HCV vaccine development have highlighted the importance of nAb and cellular responses for protection against HCV [3], [4], [30]–[38], and a balanced T cell response and broad-spectrum nAb activity is ideal for HCV vaccine development [3], [4]. However, the development of a B cell-based vaccine that elicits a strong and broad nAb response is a difficult task due to the high genetic variability of HCV [16]. HCV VLPs consisting of the HCV core, E1, and E2 proteins protected against HCV infection in mice and chimpanzees, suggesting that these virion structural proteins play an important role in HCV clearance [35], [39]. Clinical data and animal experiments show that T cell immunity against the non-structural protein NS3 was related to recovery from HCV infection [3, 4, 21, and 40]. HCV-nAb studies were limited by the technology available before 2003, and studies of vaccines based on envelope proteins showed poor neutralisation capacity against a challenge with heterologous HCV genotypes [3], [4]. MuLV-derived HCVpps combined with rAd5 induced higher cross-nAb levels against multiple HCV genotypes in mouse and macaque models than any previous effort [41]. LVs can infect not only dividing but also non-dividing and resting cells, and so have greater potential for vaccine and gene delivery than MuLV vectors [42]–[45]. Furthermore, IDLVs surmount the risk of random integration; thus, IDLVs have many merits for vaccine delivery [45]. Thus, in this study we used IDLVs bearing HCV envelope proteins and NS3 mRNA to bring together as many desirable characteristics as possible for HCV vaccine design.

Our previous data indicated that vaccination with rAd5-CE1E2 induced significant CMI in mice; however, no nAbs were detected [21].Now our results indicated that immunisation with IDLV-HCVpps elicited strong humoral immunity and cross-nAbs based on different genotype of HCVpp; however, no T cell response was detected by IFN-γELISPOT assays. In contrast, priming with rAd5-CE1E2 and boosting with IDLV-HCVpps resulted in a robust CMI against E1, E2, and NS3, which increased with the dose and number of immunisations. This indicates that IDLV-HCVpps have the capacity to induce potent T cell responses. Preclinical studies in chimpanzees showed that the addition of CpG or MF59-adjuvanted gpE1/gpE2 significantly enhanced antibody titres as well as T-helper cell responses to the vaccine [34], [37]. We also compared the antibody responses when IDLV-HCVpp immunogens were coupled with or without adjuvant combinations (Al and CpG ODN). The addition of a combined adjuvant (Al [OH] 3+CpG) had a limited effect on the humoral immunity induced by IDLV-HCVpps at higher boost doses (data not shown).

**In conclusion,** we developed and validated a novel IDLV-HCVpp platform that pseudotyped an array of three heterologous neutralising target proteins in their proper conformation, and which harboured critical T cell epitopes within IDLV virions. Our results show that the IDLV-HCVpps alone induced a humoral immune response, including IgGs to E1, E2 and cross-nAbs. Furthermore, the protective immunity, and particularly the broad cross-neutralising capacity or multi-antigen specific T cellular immunity, could be enhanced by the use of an optimal regimen (e.g., cocktail protocols or a prime-boost strategy). To our knowledge, this is the first report of the application of an IDLV platform to HCV vaccination and the optimisation of an immunisation regime in mice. A potential added value of IDLVs as a delivery vehicle is the ability to induce a significant gag-specific immune response for protection against HIV infection, in addition to the transgene-specific response. Based on our report of the potential of IDLV-HCVpps in mouse models, additional studies on and the validation of IDLV-HCVpps immunization and prime-boost strategy, including comparisons with other vaccine protocols and use in non-human primate models, are warranted. Furthermore, the present study establishes a potential powerful approach for elicitation both broad-spectrum nabs activity and balanced T cell response against viral diseases, especially suitable for highly variable pathogens (such as HIV and influenza).
